# Frequency of placental malaria and its associated factors in northwestern Colombia, pooled analysis 2009–2020

**DOI:** 10.1371/journal.pone.0268949

**Published:** 2022-05-24

**Authors:** Jaiberth Antonio Cardona-Arias, Jaime Carmona-Fonseca

**Affiliations:** 1 School of Microbiology, University of Antioquia, Medellín, Colombia; 2 “Grupo de investigación César Uribe Piedrahíta”, Faculty of Medicine, University of Antioquia, Medellín, Colombia; University of Khartoum, SUDAN

## Abstract

Knowledge about placental malaria (PM) is insufficient in the world, and incipient in Colombia where studies are few and recent. In this country, PM has been reported by *Plasmodium viva*x, *Plasmodium falciparum*, and mixed infection. The objective was to determine the frequency of PM and its associated clinical-epidemiological factors in mothers and neonates in northwestern Colombia, 2009–2020. A Retrospective pooled analysis with 602 placentas captured in five investigations. The diagnosis of PM was made with thick blood smear (TBS) and qPCR. The groups with and without PM were compared using the Chi-square test, Mann-Whitney test, and crude and adjusted prevalence ratios in a log-binomial model. The prevalence of PM was 27.7% with 92% (155/167) of submicroscopic cases; 41.3% by *P*. *vivax*, 44,3% by *P*. *falciparum*, and 14.4% by mixed infections. In the multivariate adjustment, PM was associated with the diagnosis of congenital malaria, low neonatal weight, gestational malaria, maternal anemia, previous malaria during pregnancy, and age between 25–43 years. This research is the investigation with the largest number of subjects for studying PM in Colombia, in the ecoepidemiological zone that produces more cases of malaria per year, finding a high prevalence of submicroscopic PM that caused serious maternal (anemia) and neonatal (congenital malaria and low neonatal weight) effects. The results show limitations in the timely diagnosis and treatment, given that the epidemiological surveillance program in Colombia is based on thick blood smear, which generates a substantial underestimation of the magnitude of PM, with serious effects and clinical risks. It is urgent to demand that the health authorities adopt measures such as prenatal control visits as soon as the pregnancy begins, monthly implementation of TBS, and active search for infected pregnant women in their homes and workplaces.

## Introduction

Malaria associated with pregnancy includes three clinical-epidemiological events: *i)* gestational malaria or infection demonstrated in maternal peripheral blood, *ii)* congenital malaria or infection in the neonate by transplacental transmission, and *iii)* placental malaria (PM) corresponding to the presence of *Plasmodium* spp. in this organ. There is little information about these three events. Researches on PM presents a predominance of immunological or histological themes, with few publications on epidemiological issues [1–5]; for example, a search in Scielo.org with the syntax “(*ab*:(*placental malaria*))” only generates six results and in Pubmed with “*placental malaria* [*Title/Abstract*]” only 13 publications.

Regarding immunology and other processes affected by PM, it is clear that cytokines and mediators of physiological processes (apoptosis, hypoxia, inflammation, angiogenesis) show intense alteration with either of the two plasmodial species and that in the immune response of PM, the role of immune cells is crucial but poorly understood [6–10].

The histopathology of PM has been reported in few manuscripts for several decades, which have not had a standard protocol to identify and quantify the magnitude of the lesions. More than 90% of the publications are focused on Africa and deal only with PM caused by *P*. *falciparum*, even though there are arguments to affirm that *P*. *vivax* also produces PM [10–16]. Furthermore, it was recently reported that placental histological changes and PM are independent risk factors for preeclampsia, especially in primiparous [17].

Another study reported that the number of *P*. *falciparum* infections in pregnancy is a predictor of PM with variations according gravidity; in primigravidae with PM, earlier asymptomatic infections were more frequent, whereas in multigravidae were detected later in pregnancy [18]. Furthermore, in Africa, it was reported that primigravidae with less than four antenatal care visits had the highest risk of PM [19]. Among 1115 women with histopathology and DNA PCR, 8% had histology-positive for PM, and 12% submicroscopic PM by *P*. *falciparum*; The risk of sub-microscopic PM was significantly higher in women who did not use mosquito prevention methods (bed nets, fumigation, or mosquito coils) and anemic women; The histology-positive for PM increased the risk of small-for-gestational-age births, and PM was not associated with low birth weight nor prematurity [20].

In Colombia 91.2% of the territory, with 749 municipalities and 9,734,271 inhabitants, has active malaria transmission. Transmission is localized in some areas, with 96.7% of the disease burden in the Pacific and Uraba-Bajo Cauca-Sinu-San Jorge regions (the latter correspond to the region where this study was carried out, where *P*. *vivax* predominates [21].

Besidesa, in Colombia, there are few publications on PM, and they concentrate on histopathological and immunological issues [8–10, 22]. Epidemiological studies on PM have reported frequencies between 0.3% and 12.8% with TBS (thick blood smear), and 2.7% to 57.3% with PCR (polymerase chain reaction); These studies have focused on the comparison of diagnostic tests, the detection of asymptomatic and submicroscopic infections (positive with PCR and negative with TBS) [8, 16, 23–25]. Knowledge about maternal and neonatal factors and the history of malaria potentially associated with PM has been less explored. In this sense, two investigations, one with 95 placentas and the other with 179, found no association of PM with maternal and gestational age, parity, number of pregnancies, history of malaria, or with weight, height and head circumference at birth. One of the investigations did not report an association with hemoglobin, and the other did, with a higher percentage of anemia in the group with PM. [8, 26].

Based on the above, there is a need for further studies on PM’s frequency and associated factors. Therefore, the objective of this research was to determine the frequency of PM and its associated factors in northwestern Colombia, grouping five investigations conducted between 2009 and 2020. These investigations followed the same diagnostic procedures for PM and applied the same forms to collect clinical-epidemiological information.

## Materials and methods

### Study type and location

It is a retrospective pooled analysis with placental samples and information collected in northwestern Colombia, a region made up of 21 municipalities in the department of Antioquia (specifically the areas of Urabá and Bajo Cauca) and four municipalities in the department of Córdoba (upper basins of the Sinú and San Jorge rivers). This region reports about 60% of the country’s malaria cases, with an annual parasite rate> 25/1,000 exposed. Malaria can be contracted throughout the year in this region, meaning that its transmission is stable and mesoendemic (plasmodial infection between 11% -50%) [27]. According to official data from Colombia (obtained from the National Administrative Department of Statistics DANE (for its acronym in Spanish)), the inhabitants of the study region in 2019 were 1,114,000, all exposed to malaria, which included approximately 5,414 pregnant women [28, 29].

### Study population and sample size

In this study, 602 placentas of women with spontaneous and vaginal delivery, captured between 2009 and 2020, were analyzed. No sample size calculation was made because this total corresponds to the placentas of women who agreed to participate in the research projects of the group “Salud y Comunidad César Uribe Piedrahíta”, and fulfilled the selection criteria.

The inclusion criteria were being a permanent resident of the area for at least a year before the beginning of the study, no history of preeclampsia/eclampsia, high blood pressure, diabetes, HIV or other sexually transmitted infections, TORCH (Toxoplasmosis, Other agents, Rubella, Cytomegalovirus, Herpes simplex), voluntarily participation in the study and signing of the informed consent or assent. The exclusion criteria were not having TBS or qPCR results for the diagnosis of PM, preterm delivery (between weeks 22–36) or with abnormalities, and being under antimalarial treatment for the two weeks before the sample collection. The elimination criteria corresponded to withdrawal during the execution of the projects due to being diagnosed with some morbid event or complication during pregnancy or the withdrawal of consent.

### PM diagnosis

To establish the diagnosis of PM, TBS and PCR were used, and the existence of PM was accepted if one or both tests were positive (without PM when both tests were negative). TBS was performed in all five investigations, as established by the WHO, and qPCR was applied for molecular diagnosis [8, 30].

### Data collection and quality control

On the day of delivery, placental blood samples were taken. In some cases, maternal, neonate, and umbilical cord peripheral blood was also collected (depending on the pregnant woman and the newborn’s medical condition and the sample collection facilities of the place of delivery). Slides were made for the microscopic diagnosis of malaria using TBS, and Whatman filter paper circles # 3 were impregnated for DNA extraction with the Saponin-Chelex method and subsequent molecular diagnosis of plasmodial infection using qPCR [8, 10]. For the microscopic diagnosis of PM, two samples were taken for pathology, one from the insertion area of the umbilical cord and another from the middle area of the placenta. From the space generated by the extraction of these two parts of tissue, blood was taken to perform the TBS; In this way, a TBS was obtained from the middle area of the placenta and another from the insertion area of the cord (placentas with one or both positive samples were defined as PM positive) [8, 10, 30].

Additionally, a form was filled out with the following maternal and neonatal variables: age, number of pregnancies and deliveries; self-report of malaria during the current pregnancy; use of mosquito net; diagnosis of gestational malaria using TBS and qPCR; maternal hemoglobin (anemia with hemoglobin <11g/dL); the number of abortions, fetal deaths and stillbirths; as well as weight, height, head circumference, and Apgar score at birth (SI 1).

Quality control was guaranteed through training and standardization of fieldwork, internal quality control in the laboratory, implementation of the manufacturer’s instructions for the tests, double data entry, and appearance and content validity of the maternal-neonatal variables extraction form.

### Statistical analysis

The qualitative maternal-neonatal variables were described with absolute (n) and relative (%) frequencies, while the quantitative variables with median, interquartile range, and range, given that they did not fulfill the assumption of normality (according to Lilliefors-corrected Kolmogorov-Smirnov).

The general frequency of PM, specific frequency by diagnostic test and plasmodial species, and frequency of submicroscopic PM were calculated, all with their 95% confidence interval, and were compared with the Z statistic.

The comparison of PM with the categorical variables was carried out with the Chi-square test for trend for ordinal variables and Pearson’s Chi-square test for nominal variables; The comparison with the continuous variables were made with the Mann-Whitney U test. For the associated variables, the strength of the association was determined with prevalence ratios and their 95% confidence intervals. A multivariate generalized linear model was performed with the logarithm and binomial family transformation (log-binomial) to identify the variables with the most significant explanatory potential for PM [31, 32].

All analyzes were carried out in SPSS 27.0, taking p values less than 0.05 as significant.

### Ethical aspects

The guidelines of the Declaration of Helsinki, Resolution 8430 and Resolution 1995 of Colombia were applied. The study was classified as minimal risk and was endorsed by the Ethics Committee of the SIU (Sede de Investigación Universitaria), University of Antioquia, Minutes 21-101-961. All pregnant women signed the informed consent (of legal age) or assent (under 18 years of age), obtained in writing, with the signature of the pregnant woman, a witness (external to the research group) and the member of the health team who explained the content of the informed consent-assent. According to Colombian law, adolescents (over 10 years old) can sign the informed assent without requiring consent from parents or guardians, as was the case of the adolescent pregnant women included in this research (informed consent were not obtained from the parent/guardians of pregnant women aged 13–17 years). All the data used in this research were anonymized by someone outside the research team (health personnel who participated in the collection of the information), so that the authors of this manuscript had access to a database with codes (without name, or identification number, or other information that will show the identity of the pregnant women), to guarantee the confidentiality of the data.

## Results

The frequency of PM was 27.7%, no statistical differences were found between the proportion of cases due to *P*. *vivax* (11.5%) and *P*. *falciparum* (12.3%) (Statistical Z = 0.356 p = 0.722); 92% (155/167) of the total cases detected were submicroscopic PM (Table 1).

**Table 1:** Frequency of placental malaria according to species and diagnostic test.

Specie	TBS		qPCR		Submicroscopic PM	
	n	% (CI95%)	n	% (CI95%)	n	% (CI95%)
*P*. *vivax*	9	1.5 (0.4–2.6)	69	11.5 (8.8–14.1)	60	10.0 (7.5–12.4)
*P*. *falciparum*	4	0.7 (0.2–1.7)	74	12.3 (9.6–15.0)	70	11.6 (9.0–14.3)
*Mixed*	0	0	24	4.0 (2.3–5.6)	24	4.0 (2.3–5.6)
Total	13	2.2 (0.9–3.4)	167	27.7 (24.1–31.4)	154	25.6 (22.0–29.2)

The highest proportion of pregnant women were adolescents or young women (61.6%), with one or two pregnancies (54.8%), multiparous (44.6%), and who reported using mosquito nets (62.8%). A frequency of maternal anemia of 29.9%, a self-report of previous malaria during the current pregnancy of 22.6%, and a diagnosis of gestational malaria of 23.1% (78% of submicroscopic cases) were found. Additionally, 15.4% had abortions, 1.8% stillbirths, 6.0% low birth weight, and there were no fetal deaths (Table 2).

**Table 2:** Obstetric and neonatal characteristics in the studied group and disaggregated between women with and without placental malaria (PM).

Variables	Categories	Without PM ^a^	With PM ^b^	Total ^c^
**Maternal variables**		**Proportion % (n)**		
**Age group (years)**	Adolescents (13–19)	26.9(117)	26.3(44)	26.7(161)
	Youth (20–24)	35.4(154)	33.5(56)	34.9(210)
	Adults (25–43)	37.7(164)	40.1(67)	38.4(231)
**Pregnancies**	One	32.0(135)	28.4(46)	31.0(181)
	Two	24.9(105)	21.0(34)	23.8(139)
	Three	15.6(66)	25.9(42)	18.5(108)
	Four to ten	27.5(116)	24.7(40)	26.7(156)
**Deliveries**	Nulliparous (0)	26.7(101)	54.4(37) ^d^	30.9(138)
	Primiparous (1)	24.6(93)	23.5(16)	24.4(109)
	Multiparous (2–10)	48.7(184)	22.1(15) ^d^	44.6(199)
**Gestational malaria diagnosis**	Total	14.3(60)	55.8(63) ^d^	23.1(123)
	Submicroscopic	11.6(49)	41.6(47) ^d^	18.0(96)
	Positive with PCR-GG	2.6(11)	14.2(16) ^d^	5.1(27)
**Others**	Use of mosquito net	62.0(217)	70.3(26)	62.8(243)
	Malaria in this pregnancy	14.5(63)	43.7(73) ^d^	22.6(136)
	Maternal anemia	22.3(63)	45.3(63) ^d^	29.9(126)
		**Neonatal variables % (n)**		
**Congenital malaria**	Total	3.8(13)	35.8(39) ^d^	11.6(52)
	Submicroscopic: peripheral blood	1.0(2)	11.5(3) ^d^	2.2(5)
	Submicroscopic: cord blood	5.5(11)	41.4(36) ^d^	16.3(47)
**Others**	Abortions	15.0(52)	19.4(7)	15.4(59)
	Stillbirths	1.7(6)	2.8(1)	1.8(7)
	Low Neonatal Weight (≤2.500g)	4.2(17)	10.7(17) ^d^	6.0(34)
**Continuous neonatal variables**		**Median (Interquartile range)**		
Mother’s age (years)		22(19–28)	22(19–29)	22(19–28)
Hemoglobin (g/dL)		11.8(11.0–12.5)	11.0(10.2–12.0) ^e^	11.5(10.7–12.3)
Birth weight (kg)		3.2(3.0–3.5)	3.0 (2.7–3.3) ^e^	3.1(2.9–3.5)
Height at birth (cm)		50(49–52)	49 (48–51) ^e^	50(49–52)
Head circumference (cm)		34(33–35)	33 (32–34)^e^	34(33–35)
5-minutes Apgar score		9(9–10)	9(8–10)	9(9–10)

^a^ The numerator is the subjects that meet each characteristic shown in the rows, taking as denominator the total placenta Negative for malaria.

^b^ Same numerator as above, taking as denominator the total placenta Positive for malaria.

^c^ The valid percentage was taken (excluding the missing for each variable from the denominator).

^d^ Statistically significant differences (Chi2 p<0.01) were found in the group with and without placental malaria (PM).

^e^ Statistically significant differences (Mann Whitney U p<0.01) were found in the group with and without PM.

The frequency of PM was statistically higher in nulliparous women (26.8%), women with a diagnosis of gestational malaria (51.2%), women who reported having malaria previously during the current pregnancy (53.7%), women with maternal anemia (50.0%), and among neonates with congenital malaria (75.0%) and low birth weight (50,0%). In addition, a statistically lower median was found in the PM group for hemoglobin and weight, height, and head circumference at birth, compared to the group without infection (Table 3).

**Table 3:** Frequency of placental malaria according to obstetric and neonatal conditions.

Categorical variables		Placental malaria n (%)	Prevalence ratio (CI95%)
Deliveries	Nulliparous (0)	26.8 (37)	3.6 (2.0–6.2)**
	Primiparous (1)	14.7 (16)	2.0 (1.0–3.8)*
	Multiparous (2–10)	7.5 (15)	1.0
Gestational malaria diagnosis	Positive	51.2 (63)	4.1 (3.1–5.7)**
	Negative	12.2 (50)	
Malaria in this pregnancy	Positive	53.7 (73)	2.7 (2.1–3.4)**
	Negative	20.2 (94)	
Maternal anemia	Positive	50.0 (63)	2.0 (1.5–5.5)**
	Negative	25.7 (76)	
Diagnosis of Congenital Malaria	Positive	75.0 (39)	4.2 (3.3–5.5)**
	Negative	17.7 (70)	
Low Neonatal Weight	Positive	50.0 (17)	1.9 (1.3–2.7)**
	Negative	26.9 (142)	

*p<0.05.

**p<0.01.

The frequency of PM was not associated with the age group (Chi^2^ = 0.585), use of mosquito net (Chi^2^ p = 0.322), number of abortions (Chi^2^ p = 0.485), or stillbirths (Chi^2^ p = 0.659); There was no association between the continuous variables and the APGAR score (Mann-Whitney U p = 0.434), age of the mother (Mann-Whitney U p = 0.364), number of pregnancies (Mann-Whitney U p = 0.398), number of stillbirths (Mann-Whitney U p = 0.659), and fetal deaths (Mann-Whitney U p = 0.999).

In the multivariate adjustment, the PM showed a greater strength of association with the diagnosis of congenital malaria (25.3 times greater compared to the group without PM), low birth weight (11.2 cases in the group with PM for each case found in the group without PM) and gestational malaria (8.5 times more compared to the group without PM). Other associated variables were maternal anemia, suffering malaria previously during pregnancy, and being an adult (Table 4).

**Table 4:** Potential explanatory factors for placental malaria.

	B	Wald Chi-square	Likelihood ratio (IC95%)
Congenital malaria (Yes/No)	3.2	34.5	25.3 (8.8–73.0)*
Low Birth Weight (Yes/No)	2.4	8.0	11.2 (2.9–43.2)*
Gestational malaria (Yes/No)	2.1	27.9	8.5 (3.9–18.5)*
Maternal anemia (Yes/No)	1.3	10.8	3.8 (1.7–8.1)*
Malaria in current pregnancy (Yes/No)	1.2	7.7	3.5 (1.5–8.2)*
Age group (25-43/13-24 years old)	1.0	7.7	2.8 (1.3–5.9)*

*p<0.01.

## Discussion

The frequency of PM was 27.7%, which is a very high value and represents serious risks of PM affecting maternal, fetal, and neonatal health; furthermore, it is a different frequency from that reported in previous studies in Colombia, which have reported values between 2.7% and 57.3% (with an average of NN%) employing nested or in real-time PCR [8, 23–25]. This heterogeneity could be based on several aspects related to the design of the studies, technological drift, varying malaria endemicity or intensity of malaria transmission, conditions of the pregnant women (symptom, immunity, use of mosquito nets, insecticides, self-medication), or particularities of the locations studied (conditions of life, or social, economic, cultural, and political determinants of health).

Together these aspects demonstrate the need to increase the number of studies on this subject, considering the number of PM aspects that are unknown in Colombia (a situation that can be extrapolated to many endemic regions of the world), as well as the impossibility of inferring or extrapolating the evidence generated in previous studies, even within the same country.

The submicroscopic PM was widely dominant (92% of the total cases detected), demonstrating the high proportion of false negatives with TBS. Consequently, not capturing cases on time, nor providing them with treatment; the risk of it becoming a severe form of the disease, generating deleterious effects on neonatal health; and a delay in the control or pre-elimination goals outlined in some official documents [16, 25, 33–35].

The specific frequencies by species were statistically equal (*P*. *vivax* with 41.3% of the cases, *P*. *falciparum* 44.3%, and mixed infection 14.4%), which is striking because, in previous studies on gestational malaria in the same ecoepidemiological area, P. vivax has predominated widely (65–70%). These results would denote a kind of independence between both compartments (maternal and placental peripheral blood) and show the importance of improving etiological and physiological studies on the roles of the placenta in the permanence or control of infections by different plasmodial species. Besides the similarity in the frequency of both species, previous studies on PM in Colombia have also described similar histopathological patterns for *P*. *vivax* and *P*. *falciparum* [10, 26].

The maternal variables associated with PM in the multivariate adjustment were: being an adult, which could reflect (be a proxy for) a longer exposure time due to living in areas without adequate control of transmission; suffering from malaria previously during the current pregnancy; and the diagnosis of gestational malaria, which would demonstrate an independent effect of each previous diagnosis of malaria on PM; showing the need to diagnose and treat each case on time, even from the preconception period, in order to avoid PM and its risks to fetal and neonatal health [33].

In the multivariate adjustment, other associated factors that function as consequences of PM were the increased risk of maternal anemia, congenital malaria, and low birth weight, consistent with previous publications on the effects of malaria associated with pregnancy [33]. However, it disagrees with the previous evidence from Colombia, in which these associated factors had not been identified [8, 26], possibly due to the low statistical power of the comparisons given that the central objective in these investigations was not the identification of associations. This difference demonstrates the importance of this study based on a large sample size that improves the statistical power while making visible negative impacts of PM that are poorly documented in Colombia. These associated factors and effects of PM have also been described in previous studies on gestational malaria [16, 25, 33, 34], from which it could be inferred that the placenta is a crucial point to identify the physiological mechanisms that connect the maternal peripheral blood infection with neonatal outcomes or to show that placental histophysiological processes are not determinant for the protection of the gestation product.

In this sense, previous studies have described parasite sequestration by the placenta that alters the functional structure (increase in syncytial nodes and necrosis) [2], the inflammatory immune response (mainly increase in IL-10 and TNF-α) [1], and the exchange of substances at the maternal-fetal interface, which would explain outcomes such as fetal death, low birth weight, premature delivery and short stature [35–40]. Furthermore, PM increases the risk of vertical transmission of other infections such as HIV [41, 42]. Therefore, the treatment of malaria during pregnancy would result in the control of its clinical effects, and indirectly, it would reduce the risk of other congenital infections.

## Conclusion

This research is the investigation with the largest number of subjects to study PM in Colombia, in the ecoepidemiological zone that produces more malaria cases per year in the country. The investigation found a high prevalence of submicroscopic PM that caused serious maternal (anemia) and neonatal effects (congenital malaria, low neonatal weight). The results show limitations in the timely diagnosis and treatment, considering that the epidemiological surveillance program in Colombia is based on thick blood smear, which generates a substantial underestimation of the magnitude of PM, with serious effects and clinical risks. Rapid diagnostic tests for malaria generally have lower sensitivity than TBS, so they cannot be proposed as an alternative; However, the country is not in a position to immediately implement molecular diagnostic tests. Therefore, it is urgent to demand from the health authorities the immediate adoption of measures such as the promotion of prenatal control visits as soon as the pregnancy begins, the monthly execution of TBS to each pregnant woman who goes to the prenatal control, and the implementation of an active search for pregnant women infected by visiting their homes and workplaces.

## Supporting information

S1 Data (XLSX)
